# Minimally invasive management of juvenile cystic adenomyoma: report of three cases

**DOI:** 10.52054/FVVO.13.3.033

**Published:** 2021-09-24

**Authors:** MR Said, H Afaneh, O Zaghmout, K Moses, OJ Young, MI Abuzeid

**Affiliations:** Department of OBGYN, Hurley Medical Center/ Michigan State University College of Human Medicine, Flint, Michigan, 1 Hurley Plaza, Flint, MI 48503, USA; Division of Reproductive Endocrinology, Department of OBGYN, Hurley Medical Center/ Michigan State University College of Human Medicine, Flint, Michigan, 1 Hurley Plaza, Flint, MI 48503, USA; IVF Michigan Rochester Hills & Flint, PC, Rochester Hills, Michigan 48307, USA

**Keywords:** Juvenile cystic adenomyosis, chronic pelvic pain, minimally invasive surgical management

## Abstract

**Background:**

Juvenile cystic adenomyosis (JCA) represents a rare form of focal adenomyosis in young women.

**Objectives:**

To determine safety and effectiveness of minimally invasive surgery (MIS for JCA).

**Materials and Methods:**

Three patients aged 16-30 years old presented with chronic pelvic pain [2016 - 2019]. Hormonal treatment failed in two cases. Cystic lesions in the myometrium (n=2), and the broad ligament (n=1) was detected on transvaginal 2D ultrasound (TV 2D US) and/or magnetic resonance imaging (MRI). The cyst was separate from the endometrium in all the cases, within the myometrium in two patients and in the right broad ligament in one case. The cystic lesions were confirmed on laparoscopy; and laparoscopic excision of the cysts with adequate repair of the myometrial beds were performed in all cases with fertility preservation. Robotic assistance was chosen in one case in an attempt to avoid injury of the fallopian tube based on the cyst location during a previous laparoscopy. The endometrial cavity was entered in one case.

**Main outcome measures:**

Absence of intraoperative complications and relief of presenting symptoms postoperatively.

**Results:**

Pathology report confirmed the diagnosis of JCA is all cases. There were no intraoperative complications. All three patients reported relief of their symptoms 6 to 8 months after surgery. No recurrence of the JCA was reported using TV 2D US in all cases.

**Conclusions:**

MIS could be the treatment of choice for patients with JCA. The technique described in our study is safe, effective, and easy to master in experienced hands.

## Introduction

Juvenile cystic adenomyosis (JCA) represents a rare form of focal adenomyosis in young women (Branquinho et al., 2012). The first cystic lesion in the literature was reported in 1908 (Brosens et al., 2015; Garcia and Isaacson, 2011). Tamura et al. (1996) described one of the earliest cases of JCA in the literature and reported an incidence of less than 1%. The pathophysiology of adenomyosis is not fully understood at this time. Some authors reported cases where the preoperative diagnosis was a noncommunicating horn of a unicornuate uterus with haematometra, and later were diagnosed as a juvenile cystic adenomyoma (Dadhwal et al., 2017; Kriplani et al., 2011). More recently, some investigators suggested that an accessory cavitated uterine mass (ACUM) should be considered in the differential diagnosis of dysmenorrhoea in adolescents (Di Spiezio et al., 2015; Jain and Verma, 2014; Panwar et al., 2020). ACUM is a rare form of a Müllerian anomaly, that can result from gubernaculum dysfunction that may lead to persistence or duplication of paramesonephric ducts forming an accessory cavity (Shah et al., 2021). This case series describes minimally invasive management of three cases of JCA.

## Materials and Methods

Three nulligravidae aged 16-30 years old presented with chronic pelvic pain (CPP) and dysmenorrhoea. Two of them had tried hormonal treatment without relief. Other causes of pain were ruled out including infectious, genitourinary and gastrointestinal. Table I illustrates the demographic data and a clinical summary of the three cases. Pre-operative imaging and surgical technique is described in each case.

### Case 1

**Figure 1:**
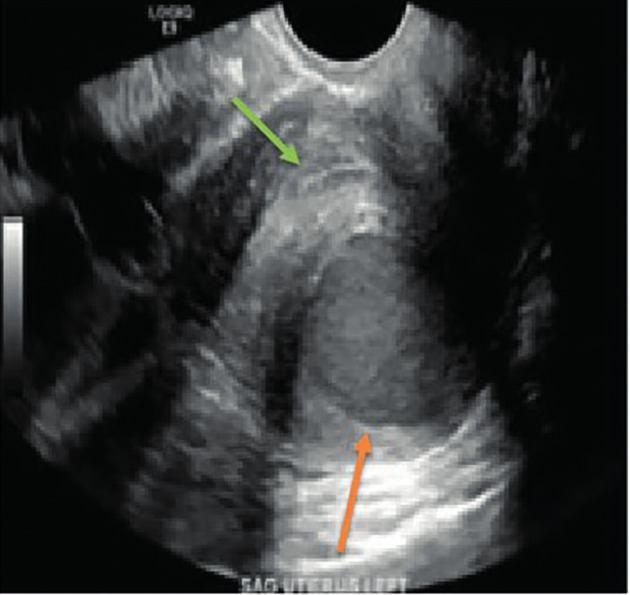
2D transvaginal ultrasound picture of a juvenile cystic adenomyosis in the posterior wall of the uterus (orange arrow). The picture is showing the endometrial strip separate from the cyst (green arrow).

**Figure 2:**
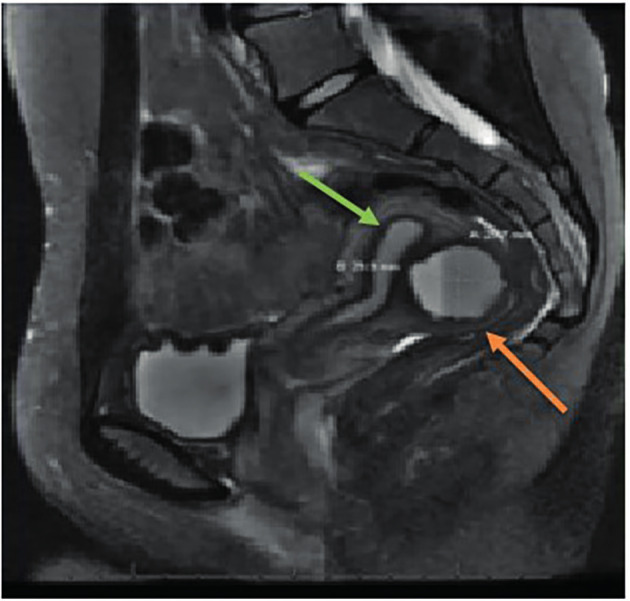
Magnetic resonance imaging picture of a juvenile cystic adenomyosis in the posterior wall of the uterus (orange arrow) of the same patient in Figure 1. The picture is showing the endometrial strip separate from the cyst (green arrow).

**Figure 3a, b, c, d:**
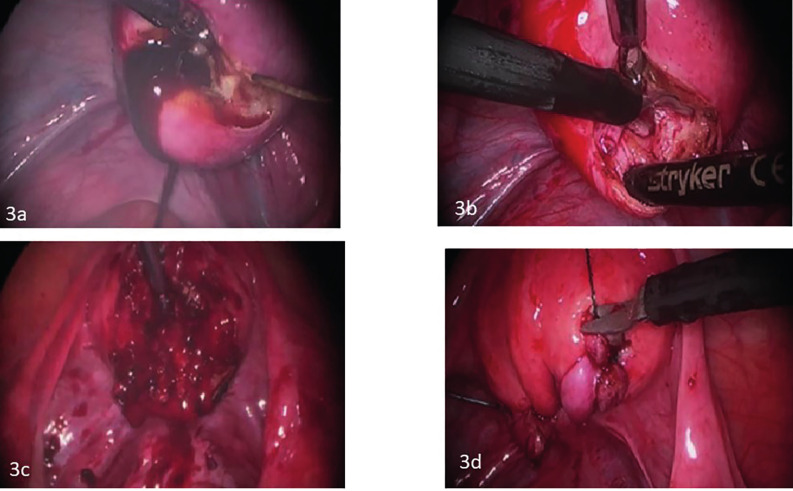
Illustrates the steps of laparoscopic excision of juvenile cystic adenomyosis in the patient in Figures 1 and 2. Figure 3a shows chocolate-like material exiting from the adenomyoma cyst on the posterior aspect of uterus. Figure 3b illustrates the removal of the cyst wall in piece meal. Figure 3c shows the adenomyoma cyst bed, following excision, with no evidence of entry of the endometrial cavity. Figure 3d shows the end result after two-layer repair of the adenomyoma cyst bed was carried using a continuous 2-0 V-lock suture. [Courtsey of Afaneh et al. Obstet Gynecol Cases Rev. 2020].

The initial pre-operative computerised tomography (CT) scan of the abdomen and pelvis showed a cystic mass separate from the endometrial cavity measuring 2.2 cm x 2.2 cm. A diagnostic laparoscopy undertaken by the referring physician revealed two small endometriotic implants on the left ovary, as well as an endometrioma of the uterus diagnosed via needle aspiration of fluid containing haemosiderin-laden macrophages and blood, with no evidence of malignancy. A hormonal intrauterine device was placed intraoperatively. About one month following her surgery, her pain recurred. The pain was severe, and was not relieved by narcotic medication necessitating immediate intervention. Repeat imaging with transvaginal (TV) 2D ultrasound (US) (Figure 1) and magnetic resonance imaging (MRI) (Figure 2) suggested recurrence of the endometrioma (3 cm x 3 cm). Decision was made for a second surgery in conjunction with a Reproductive Endocrinology and Infertility specialist. On a second laparoscopy by a reproductive surgeon, the uterine endometrioma located within the myometrium of the posterior wall of the uterus was completely excised without any difficulty. The surgical techniques that were utilised included the use of monopolar energy and sharp and blunt dissection (Figure 3a). Chocolate- like material was noted coming out of the cavity of the endometrioma (Figure 3b). The uterine cavity was not entered as determined by injecting diluted methylene blue dye transcervical (Figure 3c). The myometrial defect was closed in two-layers using 2-0 and 3-0 V-Loc suture (Medtronic, Minneapolis, MN, USA) (Figure 3d). The duration of the surgery was 90 minutes, and the estimated blood loss was approximately 40 cc.

### Case 2

**Figure 4a, b, c, d, e, f:**
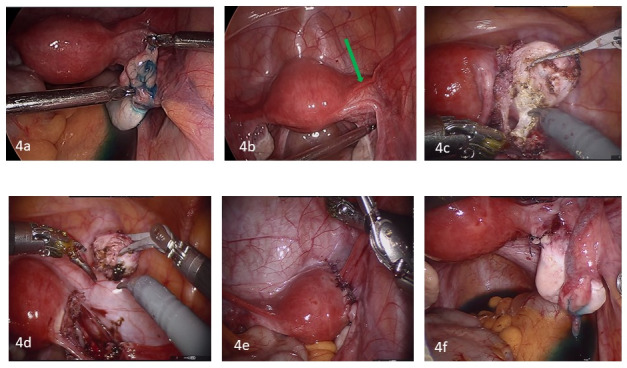
Figure 4a and b illustrate a 2 cm juvenile cystic adenomyosis mass in the right broad ligament which appeared to be continuous with the myometrium near the right cornual region and in close proximity of the isthmic portion of the right fallopian tube which was patent. Figure 4c, d show the steps of robotic assisted laparoscopic excision of the cystic mass. Figure 4e illustrates end results of the surgery after suturing the broad ligament. Figure 4f illustrates tubal patency at the conclusion of the procedure.

TV 2D and 3D US revealed adenomyosis and a cystic mass which appeared to be continuous with the myometrium. A diagnostic laparoscopy that was performed by the referring physician one year earlier showed a 3 cm mass in the right broad ligament under the proximal part of the isthmic portion of the right fallopian tube. The mass was not removed for fear of damage to the fallopian tube. Medical treatment was attempted using oral contraceptive pills for 6 months, followed by a gonadotrophin releasing hormone (GnRH) agonist for 3 months, and unfortunately both failed. During a robotic-assisted laparoscopy by a reproductive surgeon, the uterus, both ovaries, and both fallopian tubes were found to be normal; and the tubes were patent. (Figure 4a) A 2 cm right broad ligament mass with cystic consistency was stretching the fallopian tube and both round and ovarian ligaments. (Figure 4b) Healed endometriotic areas were found on the left uterosacral ligaments and pouch of Douglas. Diluted vasopressin (20 units/100cc of normal saline) was then injected between the layers of the broad ligament near the mass to decrease blood loss. Total excision of the cystic mass was performed using traction and counter traction with preservation of the right fallopian tube. (Fig. 4c, d, e, f). Estimated blood loss was 10 cc.

### Case 3

The initial TV 2D US showed an intrauterine contraceptive device in place, simple right ovarian cysts and a left complex adnexal mass 32 x 32 mm with a differential diagnosis of endometrioma versus ovarian neoplasm. MRI highlighted a 2 x 1.8 x 3 cm ovoid shaped complex cystic area adjacent to the left ovary. A follow-up TV 2D US 5 months later showed a complex left adnexal mass measuring 32 x 32 mm, and a diagnosis of an endometrioma was suggested. Tumour markers including β-HCG, alpha-fetoprotein, LDH and CA 125 were all normal. A diagnostic laparoscopy was performed by a reproductive surgeon.

**Figure 5a, b, c, d, e, f, g, h, i:**
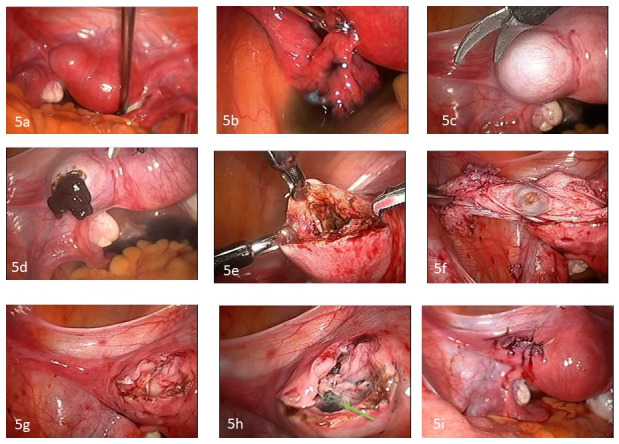
Figure 5a and b illustrate a cystic mass within the myometrium of the anterior wall of the uterus near the left cornual region in close proximity of the left fallopian tube which was patent. Figure 5c illustrates the mass after injecting diluted vasopressin. Figure 5d shows chocolate-like material exiting from the cystic mass after a transverse incision was made along the myometrium covering the cyst. Figure 5e illustrates the cyst wall which appears similar to that of endometrioma in the ovaries. Figure 5f shows the final step of stripping the cyst wall from the myometrium in one piece. Figure 5g and h illustrate the myometrial bed, after the cyst was excised, to be haemostatic and with the endometrial cavity entered in a small area (5mm) with diluted indigo carmin dye seen (green arrow). Figure 5i illustrates the end result after the myometrial bed was repaired in 3 layers using interrupted figure of eight 2-0 Vicryl sutures.

Intra-operatively, the uterus was retroverted, normal in size and contour. The ovaries were normal and both fallopian tubes normal and patent. A left 3 x 3 cm cornual mass was seen. (Fig. 5a).

The same technique of excision of the cystic mass and myometrial closure was performed as described in case 1. (Fig. 5b, c, d, e, f) However, in this case the endometrial cavity was entered in a small area during the dissection of the cyst wall from the myometrium. (Fig. 5g, h) The endometrial cavity was repaired by a one interrupted 3-0 Vicryl suture (Ethicon, Athens, GA, USA) [Figure 5i]. The estimated blood loss was less than 10 cc.

## Results

**Figure 6:**
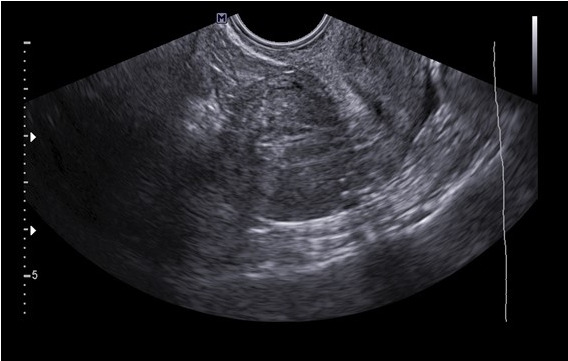
Trans-vaginal 2D US performed 8 weeks after laparoscopic excision of the JCA in the patient in Figures 1 and 2, illustrating normal appearing uterus with no evidence of recurrence of the cyst.

All the patients were discharged on post- operative day 1 and their post-operative period were uneventful. Pathology report confirmed the diagnosis of JCA is all cases. All three patients reported relief of their symptoms after 6 to 8 months after surgery with no evidence of recurrence on TV 2D US (Fig. 6).

## Discussion

The diagnosis of JCA can be challenging. We based our diagnosis in the three cases on the diagnostic criteria suggested by Takeuchi et al. (2010) who reviewed the literature and created their own diagnostic criteria for JCA including (1) age ≤ 30 years, (2) the presence of a cystic lesion measuring ≥ 1 cm in diameter and surrounded by myometrial tissue and (3) clinical symptoms of dysmenorrhea. Other nonspecific symptoms such as abnormal uterine bleeding, pelvic pain and/or infertility can be present (Shah et al. 2018). In all 3 cases in our study, imaging with TV 2D, 3D, MRI or CT scan, were used to narrow the diagnosis with the findings of a large focal, cystic lesion within the myometrium of the uterus as suggested in literature (Krentel et al., 2017).

It is important to indicate that a noncommunicating horn of a unicornuate uterus with haematometra can be misdiagnosed as a JCA during radiological studies and the correct diagnosis can only be made at time of diagnostic laparoscopy (Dadhwal et al., 2017; Kriplani et al., 2011). Therefore, JCA should be considered whenever a diagnosis of a noncommunicating horn of a unicornuate uterus with haematometra is made on radiological studies, as the planned surgical management is different.

Patients with JCA can be managed either medically or surgically. Although there are a couple of reports of successful medical treatment of JCA using a similar approach to the conservative medical therapy for endometriosis, such as using a 12- month course of GnRH agonist therapy, aromatase inhibitors or oral contraceptives in conjunction with acetaminophen and NSAIDs (Branquinho et al., 2012; Fisseha et al., 2006; ACOG Practice Bulletin 114, 2010; Ho et al., 2008), the majority of published data suggests a high failure rate of medical treatment (Akar et al., 2010; Pontrelli et al., 2015; Ball et al., 2009; Ho et al., 2009; Nabeshima et al., 2008; Grimbizis et al., 2008). Therefore, based on the current literature, one can argue that definitive surgical treatment, preferably via laparoscopy, should be the initial treatment option. However, minimally invasive surgery in such patients should only be performed by an experienced reproductive surgeon.

There are various approaches when considering surgical management for patients affected by JCA. The benefit of laparoscopic minimally invasive surgery and fertility preservation should be employed when possible depending on the surgeon expertise. The first total laparoscopic resection of a cystic adenomyoma in the literature was reported in a 27-year-old woman after unsuccessful laparotomy (Nabeshima et al., 2008). The authors reported complete resolution of the cyst associated with symptomatic relief (Nabeshima et al., 2008). Following this report, many other authors reported successful laparoscopic management of such cases (Dadhwal et al., 2017; Kriplani et al., 2011; Takeuchi et al., 2010; Ball et al., 2009; Takeda et al., 2007; Jain and Goel 2012; Strelec et al., 2019; Deblaere et al., 2019). The technique of laparoscopic excision of JCA in case 1 and case 3 of our series is similar to the techniques described by other investigators (Dadhwal et al., 2017; Kriplani et al., 2011; Takeuchi et al., 2010; Ball et al., 2009; Takeda et al., 2007; Jain and Goel, 2012; Strelec et al., 2019; Deblaere et al., 2019). In addition, there are three case reports in the literature of laparoscopic excision of a cystic mass from a unique location, namely the broad ligament (Shah et al., 2018; Trehan and Trehan, 2014; Chauhan et al., 2017). Interestingly, two of these reports named the cystic lesions an endometrioma instead of JCA (Trehan and Trehan 2014; Chauhan et al., 2017). In case 2 of our series, the cystic mass was found in the right broad ligament and was continuous with the myometrium. Although this mass was thought to be a JCA, it is possible that it was an ingrowing endometrioma in the broad ligament that became attached to the myometrium as described by other investigators (Trehan and Trehan 2014; Chauhan et al., 2017).

Another option for the laparoscopic approach is utilising the robotic system. The first case of robotic- assisted laparoscopic excision in a case of JCA was reported in 2010 and the authors suggested that the robotic-assisted approach would not only be more efficient but also be safer for patients who would like to preserve their reproductive potential (Akar et al., 2010). Although we adopted a robotic-assisted laparoscopic technique in case 2 of our series, it is too early to support the conclusions of Akar et al., 2010 based on the limited cases in the literature.

The first case of JCA in the literature, which was successfully excised via laparotomy, was described in 1996 (Tamura et al., 1996). Few other reports of successful surgical treatment of JCA via laparotomy were published (Ho et al., 2009; Dogan et al., 2008; Manta et al., 2016). In addition, a case of failed localisation of the cyst after laparotomy was published (Nabeshima et al., 2008). Subsequently, the cyst was successfully excised laparoscopically under intra-operative ultrasound guidance (Nabeshima et al., 2008).

Alternatively, hysteroscopy can be considered for lesions that are more easily accessible through the endometrial cavity. A cystic mass measuring 8 cm with loculations along the posterior wall of the uterine cavity was successfully managed using the hysteroscopic approach (Pontrelli et al., 2015). However, this unique method of surgical management should only be used by a very experienced reproductive surgeon. In addition, long- time evaluation of safety and efficacy, especially during pregnancy and delivery should be performed.

## Conclusion

Cystic adenomyoma presents both diagnostic and surgical challenges to the gynaecologist and the approach to therapy depends largely on the patient’s desire for future fertility. Our data and a review of the current literature suggest that definitive surgical treatment, preferably via laparoscopy, should be the initial treatment option. Laparoscopic minimally invasive approach appears to be safe and effective in experienced hands.
